# Direct Comparison of Cardiovascular Magnetic Resonance and Single-Photon Emission Computed Tomography for Detection of Coronary Artery Disease: A Meta-Analysis

**DOI:** 10.1371/journal.pone.0088402

**Published:** 2014-02-10

**Authors:** Lihua Chen, Xiao Wang, Jing Bao, Chengjun Geng, Yunbao Xia, Jian Wang

**Affiliations:** 1 Department of Radiology, Taihu Hospital, Wuxi, Jiangsu Province, China; 2 Department of Cardiology, Taihu Hospital, Wuxi, Jiangsu Province, China; 3 Molecular biology lab, Wuxi center for disease control and prevention, Wuxi, Jiangsu Province, China; 4 Department of Radiology, Southwest Hospital, Third Military Medical University, Chongqing, China; Scuola Superiore Sant'Anna, Italy

## Abstract

**Objective:**

To use direct comparative studies or randomised controlled trials to compare the accuracy of cardiac magnetic resonance (CMR) and single-photon emission computed tomography (SPECT) for the detection of obstructive coronary artery disease (CAD).

**Materials and Methods:**

Various databases were searched for original articles published prior to June 2013. Studies were selected that performed both CMR and SPECT in the same or randomised patients to detect CAD and that presented sufficient data to allow construction of contingency tables. For each study, the true-positive, false-positive, true-negative, and false-negative values were extracted or derived, and 2×2 contingency tables were constructed. To reduce heterogeneity, the meta-analysis was carried out in two parts: (1) coronary territory-based analysis and (2) patient-based analysis.

**Results:**

10 studies (5 studies based on patient, 4 studies based on coronary territory, and 1 study based on both) were included in the meta-analysis with a total of 1727 patients. The methodological quality was moderate. For part (1), the summary estimates were as follows: for CMR based on patient–a sensitivity of 0.79 (95% confidence interval: 0.72–0.84) and a specificity of 0.75 (0.65–0.83); for SPECT based on patient–a sensitivity of 0.70 (0.59–0.79) and a specificity of 0.76 (0.66–0.83). For part (2), the summary estimates for CMR based on coronary territory were a sensitivity of 0.80 (0.73–0.85) and a specificity of 0.87 (0.81–0.91), and the summary estimates for SPECT based on coronary territory were a sensitivity of 0.67 (0.60–0.72) and a specificity of 0.80 (0.75–0.84).

**Conclusions:**

Compared with SPECT, CMR is more sensitive to detect CAD on a per-patient basis. Nonetheless, large scale, well-designed trials are necessary to assess its clinical value on a per-coronary territory basis.

## Introduction

Coronary artery disease (CAD) is the leading cause of death in industrialized countries, and the prevalence is expected to increase worldwide [1]. The management of patients with known or suspected CAD is ideally guided by documentation of myocardial ischaemia for optimal medical therapy [2], [3].

Compared with invasive coronary angiography (CA), various noninvasive functional imaging techniques with a low rate of cardiac events such as single-photon emission tomography (SPECT), cardiac magnetic resonance (CMR), or positron emission tomography (PET) perfusion imaging are used to diagnose coronary heart disease and assess the need for revascularisation [4], [5]. In these techniques, SPECT has been widely used to evaluate myocardial ischemia. However, it exposes patients to ionising radiation, and estimates of its accuracy vary widely [6]. An increasing number of cardiac magnetic resonance (CMR) studies documented a high diagnostic performance of obstructive CAD and showed its prognostic value. Compared with SPECT, it has some advantages, such as the lack of ionising radiation, high spatial resolution, and its ability to assess multiple aspects of heart [7].

Both small or large scale, single or multi centre studies [8]–[17] have tested the accuracy of CMR compared with SPECT directly for the detection of coronary heart disease, against a reference standard of invasive coronary angiography (CA). However, the findings of these studies have been largely inconsistent. Therefore, we conducted a meta-analysis of the literature to estimate the accuracy of CMR compared with SPECT for the detection CAD. To obtain the best evidence of the diagnostic accuracy of these two methods, we restricted the scope of the meta-analysis to direct comparative studies or randomised controlled trials.

## Materials and Methods

### Criteria for Inclusion in the Study

We aimed to include studies published in any language. However, we eliminated non-English and non-Chinese articles for which a full-text translation or evaluation could not be obtained. For the detection of CAD, studies were eligible if the following criteria were met:

1. Adult patients were CAD or suspected of having CAD.
2. Both CMR and SPECT were evaluated in the same patient population (direct comparison) against an acceptable reference standard (as defined later), or patients were randomised within a study to receive either CMR or SPECT.
3. Invasive coronary angiography was the reference standard.
4. The data reported in the primary studies were sufficient for the calculation of true-positive, false-positive, true-negative, or false-negative values.

We included both prospective and retrospective studies.

We excluded studies if there were fewer than 20 patients and if multiple reports were published for the same study population. In the latter case, the most detailed or recent publication was chosen.

### Data Sources

PUBMED, EMBASE, Web of Science, and the Cochrane Library were searched independently by two observers. The search strategy included both subject headings (MeSH terms) and keywords for the target condition (coronary artery disease) and the imaging techniques under investigation (CMR and SPECT). We also included a methodological filter for studies of diagnostic accuracy. We limited our search to publications with the search term in the title or abstract of the article and a publication date no later than June 2013. Review articles, letters, comments, case reports, and unpublished articles were excluded. Extensive cross-checking of the reference lists of all retrieved articles was performed.

### Selection of Articles

Two authors initially screened the titles and abstracts of the search results and retrieved all potentially relevant reports in full. Next, they independently reviewed all relevant reports according to the predefined inclusion criteria. Disagreements were resolved by consensus or arbitration by a third author who assessed all of the involved items. The majority opinion was used for the analysis.

### Quality Assessment and Data Extraction

The same three authors extracted data from the selected reports. The methodological quality of the included studies was assessed independently by two observers using the quality assessment of diagnostic studies (QUADAS-2) tool, which was specifically developed for systematic reviews of diagnostic accuracy studies [18]–[20]. Meanwhile, the relevant data were also extracted from each study, including the author, journal name, study nation, year of publication, description of the study population, study design characteristics, magnetic field strength, type of pulse sequences, type of SPECT, and descriptions of the interpretations of the diagnostic tests.

### Meta-analysis

For each study, the true-positive (TP), false-positive (FP), true-negative (TN), false-negative (FN), sensitivity (SEN), specificity (SPE), positive likelihood ratio (PLR), negative likelihood ratio (NLR) and diagnostic odds ratio (DOR) values for the detection of lesions were extracted or derived, and 2×2 contingency tables were constructed. We calculated the sensitivity and specificity with 95% confidence intervals (CI) for each imaging test in each study. We tabulated results for studies based on per-patient separately from those for studies based on per-coronary territory. We drew forest plots to show the variation of SEN and SPE estimates together with their 95% CI. We constructed hierarchical summary receiver operating characteristic (HSROC) curves to assess SEN and SPE [21].

Exploring heterogeneity is critical for understanding the factors that influence accuracy estimates and for evaluating the appropriateness of the statistical pooling of accuracy estimates from various studies. Visual inspection of the forest plots, standard χ2-testing, and the inconsistency index (I-squared, I^2^) were used to estimate the heterogeneity of the individual studies using Stata software (Stata Corporation, College Station, TX, USA). P<0.1 or I^2^>50% suggested notable heterogeneity [22]. If notable heterogeneities were detected, the test performance was summarised using a random-effects coefficient binary regression model; otherwise, a fixed-effects coefficient binary regression model was used [23].

There are several study-level covariates that might have contributed to heterogeneity (such as study design, study characteristics, magnetic field strength) in a review. However, we did not assess factors by subgroup analyses that as in small meta-analyses this is likely to produce unreliable estimates.

With the Stata software, the presence of publication bias was assessed by producing a Deeks funnel plot and an asymmetry test. Publication bias [24], [25] was considered to be present if there was a nonzero slope coefficient (P<0.05), which suggested that only small studies reporting high accuracy had been published, and small studies reporting lower accuracy had likely not been published (P>0.1), which suggested that there was no evidence of notable publication bias.

**Figure 1:**
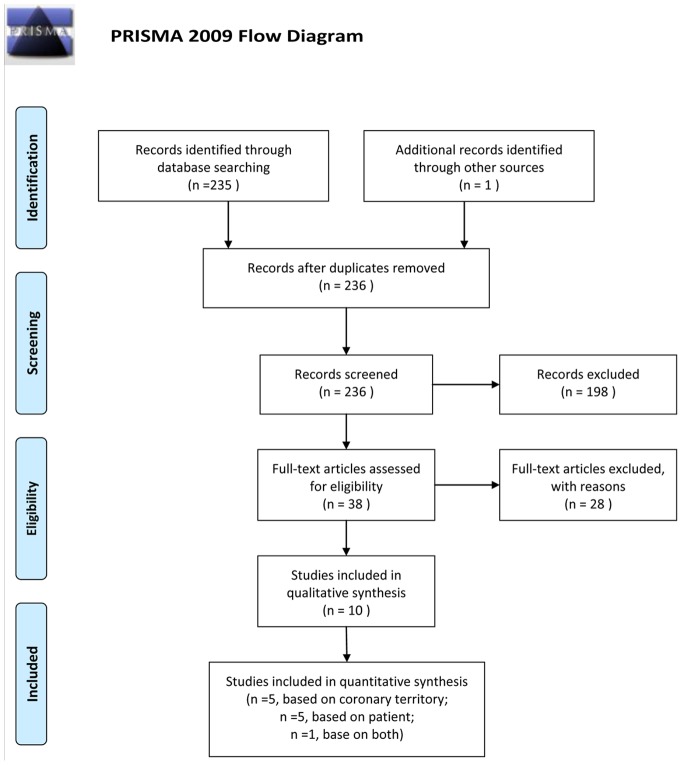
Flowchart illustrating the selection of studies.

The Preferred Reporting Items for Systematic Reviews and Meta-Analyses statement [26] was used to improve the reporting of our research (Figure 1 and Checklist S1).

## Results

**Figure 2:**
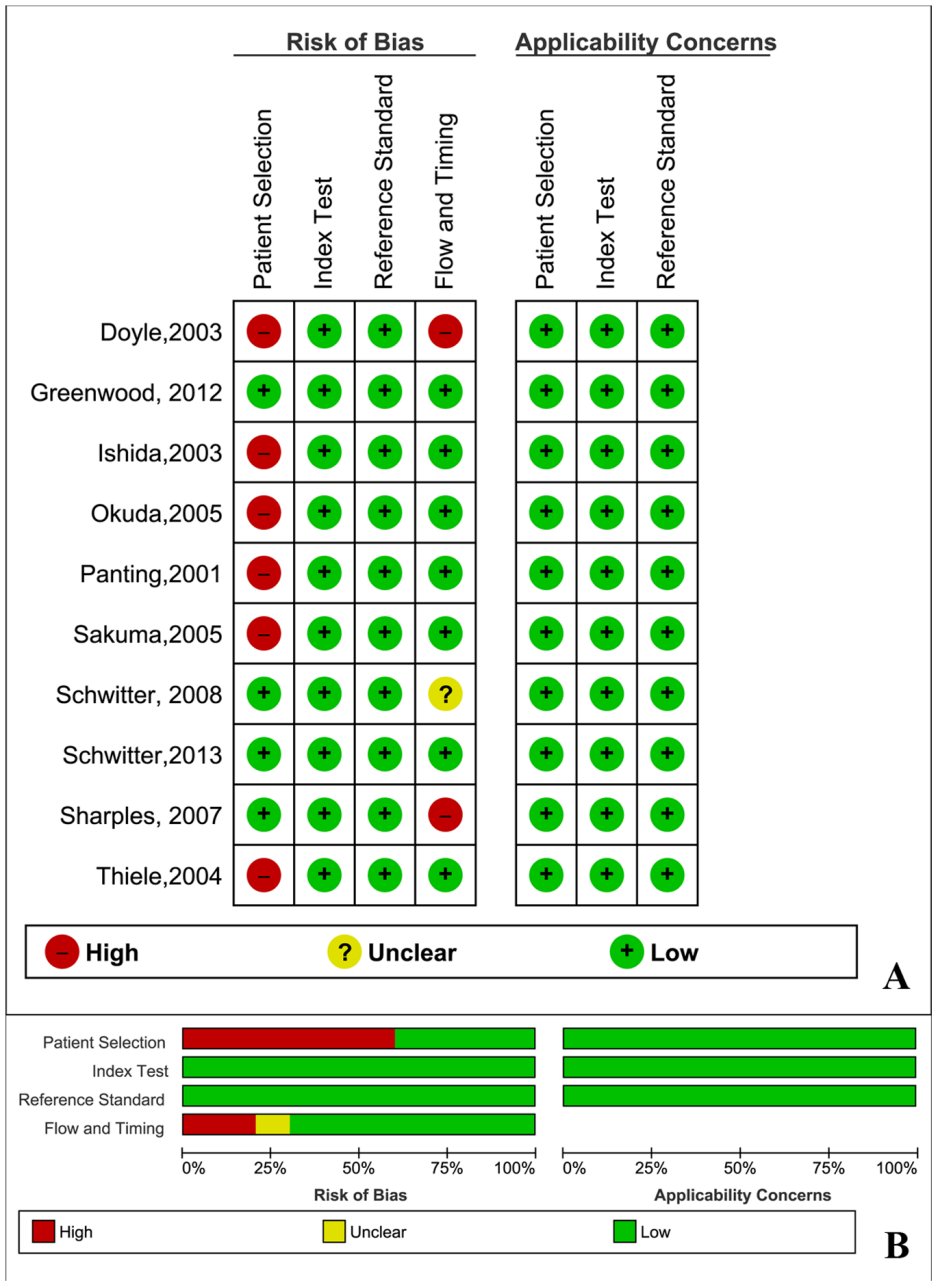
Methodological quality of the 10 included studies. A, Risk of bias and applicability concerns summary; B, Risk of bias and applicability concerns graph.

The database search initially yielded 235 potential literature citations, and one additional record was identified through a grey literature search (Figure 1). After review of the titles and abstracts, 198 of these studies were excluded because they were not relevant studies. After reading the full texts, we excluded 28 of the remaining 38 articles for the following reasons: the article lacked sufficient information to enable completion of a 2×2 contingency table, the article was not available, the article with fewer than 20 patients, or the article was not published in English. After this final screening, 10 published studies met our inclusion criteria, with 5 studies assessed base on patient, 4 studies assessed base on coronary territory (CT) and 1 study assessed base on both. Neither cost-effectiveness nor practicality was taken into consideration. The data abstracted from these individual studies are summarised in Table 1. According to QUADAS-2, the quality assessment for the 10 studies was moderate. The results of the distribution of the study design are shown in Figure 2.

**Table 1:** Characteristics of the included studies.

Study	Year	N	Excluded	Prevalence	Age(SD)	Men(%)	MRI andField	MRSequence	SPECTTracer	Dataassessment	Stressor	StenosisDefinition
Panting [16]	2001	22	0	73%	63(9)	63	Surrey 0.5T	SE-EPI	Thallium	Semi/qualitative	A	>50%
Doyle [15]	2003	199	15	14%	59(22)	80	Philips 1.5T	Gradient-echo	both	Semiquantitative	D	≥70%
Ishida [14]	2003	104	0	74%	62(12)	78	GE 1.5T	Gradient-echo	both	qualitative	D	≥70%
Thiele [13]	2004	20	0	90%	64(8)	65	Philips 1.5T	Gradient-echo	Technetium	Semiquantitative	A	≥70%
Okuda [12]	2005	33	0	97%	60(−)	88	GE 1.5T	Gradient-EPI	Technetium	qualitative	D	≥75%
Sakuma [11]	2005	40	0	52%	65(9)	70	Siemens 1.5T	Turbo FLASH	Thallium	qualitative	D	≥70%
Sharples [17]	2007	355	61	68%	62(9)	70/68	GE 1.5T	Gradient-echo	Technetium	–	A	≥70%
Schwitter [10]	2008	48	3	76%	61(11)	71	1.5T*	–	both	quantitative	A	>50%
Greenwood [9]	2012	752	76	39%	60(9)	62	Philips 1.5T	Gradient-echo	Technetium	quantitative	A	≥70%
Schwitter [8]	2013	515	90	49%	60(10)	73	1.5T*	–	both	quantitative	A	≥70%

Note: A, Adenosine; D, Dipyridamole; –, no metion; *, multi-centre with different brands.

**Figure 3:**
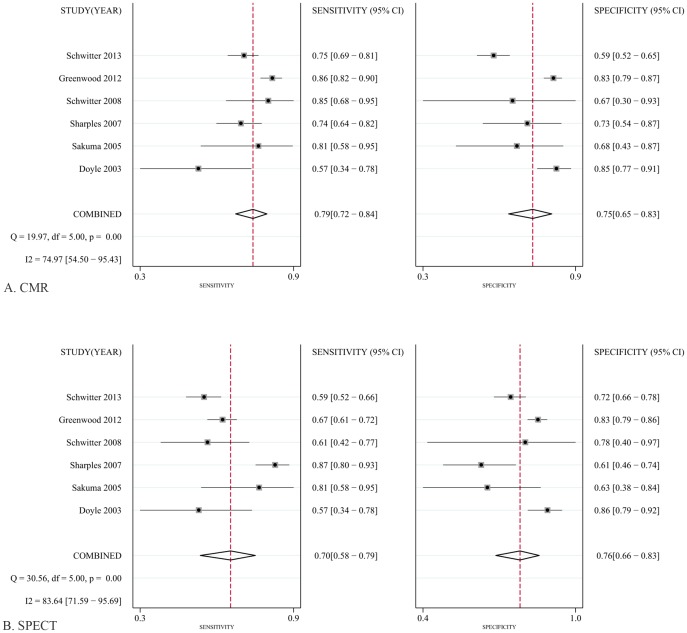
Forest plots of the SEN and SPE with corresponding 95%CIs for the detection of CAD base on patient. A, CMR; B, SPECT.

**Figure 4:**
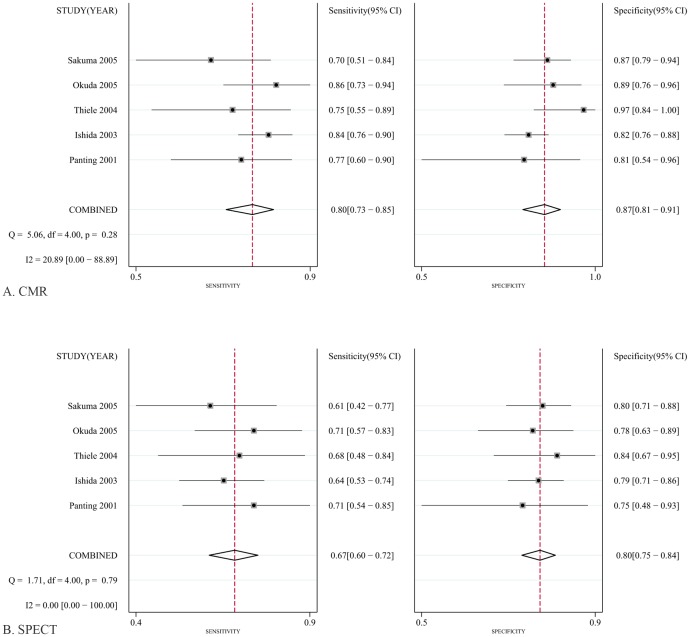
Forest plots of the SEN and SPE with corresponding 95%CIs for the detection of CAD base on coronary territory. A, CMR; B, SPECT.

**Figure 5:**
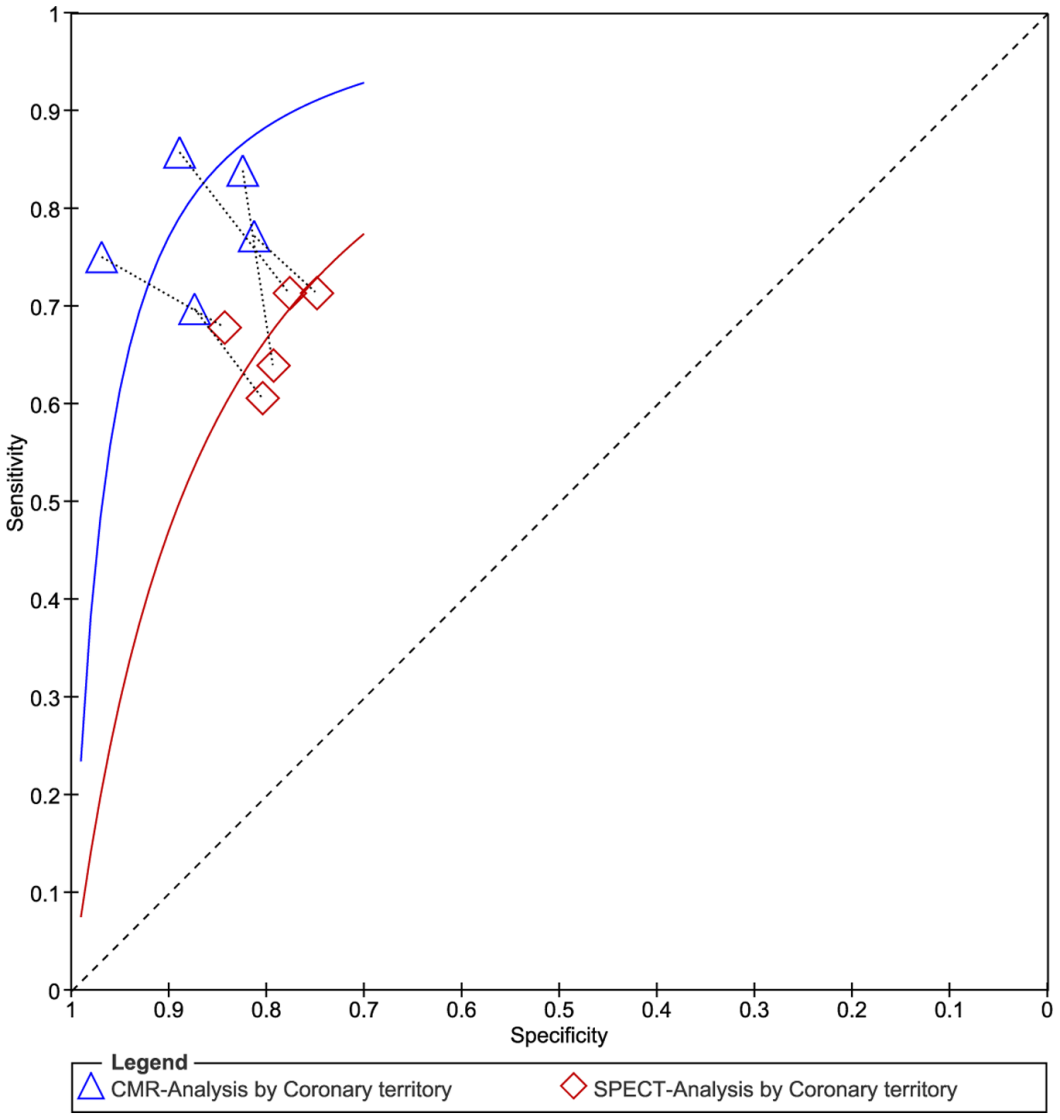
The pairs of observed values of sensitivity and specificity for CMR and SPECT to detect CAD base on coronary territory in HSROC curves.

**Figure 6:**
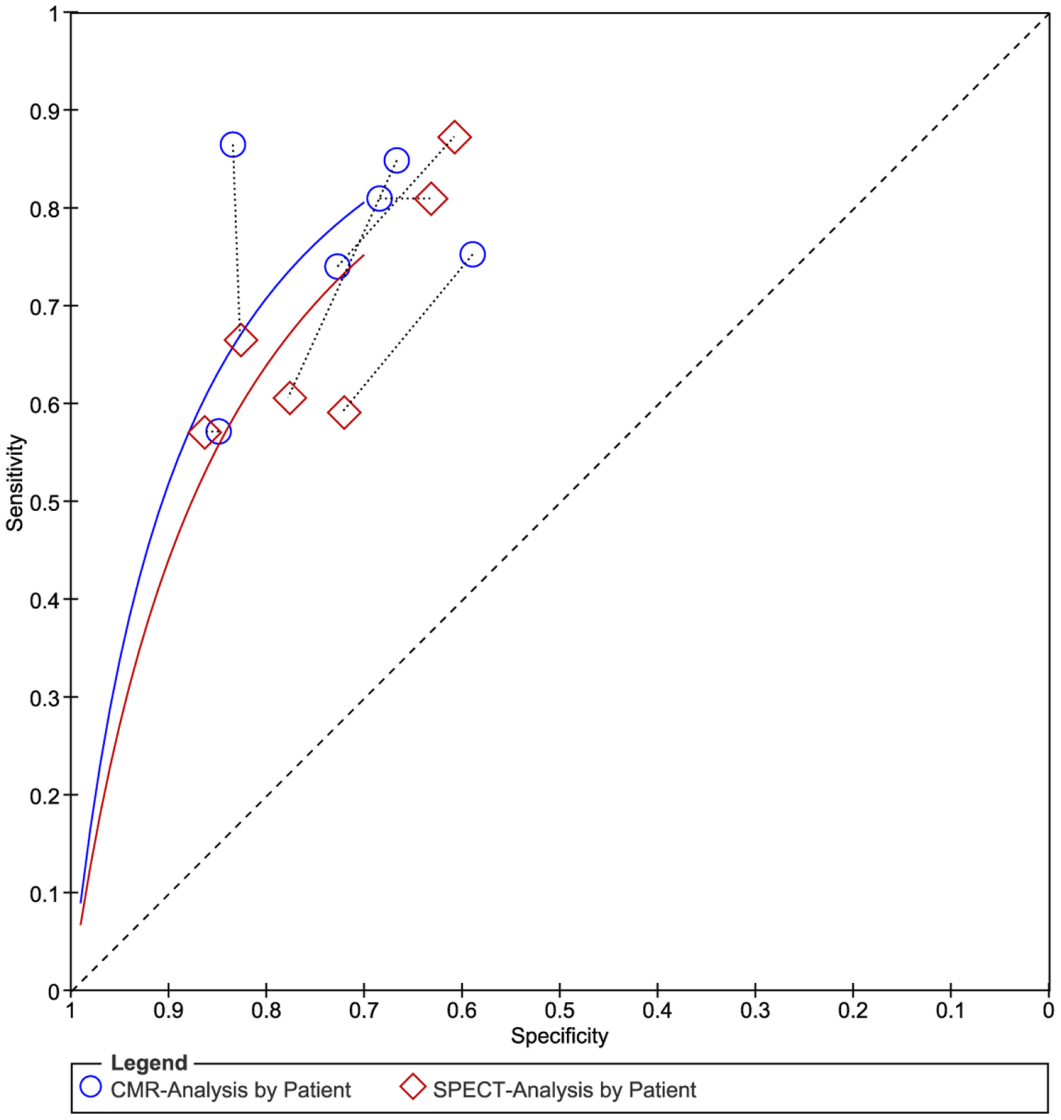
The pairs of observed values of sensitivity and specificity for CMR and SPECT to detect CAD base on patient in HSROC curves.

To reduce heterogeneity, the analysis was carried out in two parts: (a) comparison of CMR with SPECT based on coronary territory (b) comparison of CMR with SPECT based on patient. For part (1), Figure 3 shows the forest plots of the sensitivity and specificity estimates for CMR and SPECT for five studies. The pooled, weighted values (with corresponding 95% CIs) for CMR were SEN 0.91 (0.87–0.94), SPE 0.95 (0.92–0.97), PLR 16.96 (11.08–25.98), NLR 0.10 (0.07–0.14), DOR 177.14 (91.75–342.01), and AUC 0.97 (0.96–0.99). The pooled, weighted values for SPECT were SEN 0.77 (0.70–0.83), SPE 0.93 (0.84–0.97), PLR 10.32 (4.80–22.16), NLR 0.25 (0.19–0.33), DOR 41.34 (18.46–92.62), and AUC 0.88 (0.85–0.91). For part (2), Figure 4 shows the forest plots of the sensitivity and specificity estimates for six studies. The pooled, weighted values for CMR were SEN 0.92 (0.80–0.97), SPE 0.97 (0.92–0.99), PLR 29.01 (10.29–81.80), NLR 0.08 (0.03–0.22), DOR 354.19 (51.35–2443.08), and AUC 0.99 (0.97–0.99). The pooled, weighted values for SPECT were SEN 0.87(0.76–0.93), SPE 0.93 (0.86–0.97), PLR 14.01 (5.71–34.37), NLR 0.14 (0.07–0.28), DOR 98.23 (21.90–440.55), and AUC 0.96 (0.94–0.97). A summary of these results is given in Table 2. The pairs of observed sensitivity and specificity values for parts (1) and (2) are presented in HSROC curves in Figures 5 and 6, respectively.

**Table 2:** Absolute numbers of the included studies.

Study	Year	CMR				SPECT				Study design	Assess basis	Blind
		TP	FP	FN	TN	TP	FP	FN	TN			
Schwitter [8]	2013	155	90	51	129	122	61	84	158	prospective	patient	Y
Greenwood [9]	2012	230	68	36	342	177	71	89	339	prospective	patient	Y
Schwitter [10]	2008	28	3	5	6	20	2	13	7	prospective	patient	Y
Sharples [17]	2007	74	9	26	24	96	20	14	31	prospective	patient	Y
Doyle [15]	2003	12	18	9	101	12	16	9	101	retrospective	patient	Y
Sakuma [11]	2005	17	6	4	13	17	7	4	12	retrospective	patient	Y
		23	11	10	76	20	17	13	70	retrospective	CT	Y
Okuda [12]	2005	42	5	7	40	35	10	14	35	prospective	CT	Y
Thiele [13]	2004	21	1	7	31	19	5	9	27	retrospective	CT	Y
Ishida [14]	2003	109	32	21	150	55	25	31	96	retrospective	CT	Y
Panting [16]	2001	27	3	8	13	25	4	10	12	retrospective	CT	Y

**Note: CT, coronary territory.**

**Figure 7:**
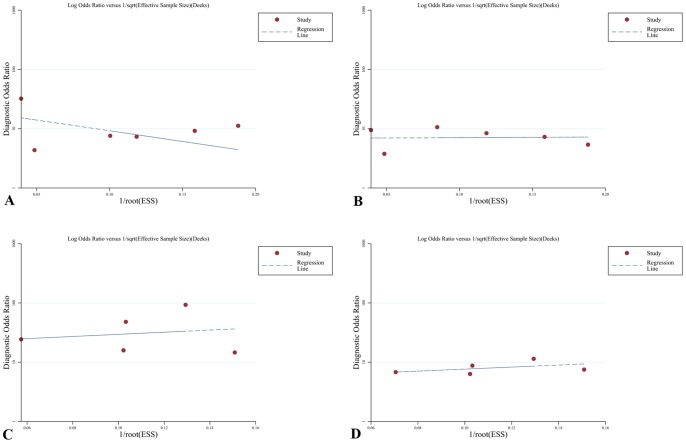
The funnel plot of publication bias. A, CMR base on patient; B, SPECT base on patient; B, CMR base on coronary territory; D, SPECT base on coronary territory.

The results of Deeks funnel plot asymmetry test (P = 0.084) showed no evidence of notable publication bias (Figure 7).

## Discussion

Although CMR has been already included in international guidelines for the non-invasive detection of coronary heart disease,it does not have the consistent findings from the previous meta-analysis and systematic review [5], [27], [28] which assessed different imaging modalities directly or indirectly, including perfusion-CMR and SPECT, for the diagnosis of CAD (Table 3). Recently, two large scale diagnostic accuracy and clinical outcome datas, MR-IMPACT II [8] and CE-MARC [9], on that have been published and also have an inconsistent finding. As direct approach is known to provide better measurements of the diagnostic accuracy of two different methods [29], we focused exclusively on direct comparative studies that evaluated both CMR and SPECT in randomised controlled or the same patients. Comparison with these previous meta-analysis and systematic review, there may be also another strength in our research: with more careful selection of articles, two direct comparative studies which missed in their researches were included in ours.

**Table 3:** Summary of meta-analysis and systematic review focused on the comparison with CMR and SPECT in the diagnosis of CAD.

Assess	Study	Search date	Modality	SEN (95% CI)	SEP (95% CI)	DOR (95% CI)	AUC (95% CI)	Comparison
Based on CT	Iwata [27]	1995–2006	CMR	0.75(0.68, 0.81)	0.89(0.85, 0.93)	24.80(14.40, 42.60)	–	direct
			SPECT	0.64(0.57, 0.71)	0.83(0.77, 0.88)	9.20(5.60, 15.10)	–	
	Our	to 2013	CMR	0.80(0.73, 0.85)	0.87(0.81, 0.91)	25.63(15.84, 41.45)	0.90(0.89, 0.93)	direct
			SPECT	0.67(0.60, 0.72)	0.80(0.75, 0.84)	7.87(5.32, 11.65)	0.80(0.76, 0.83)	
Based on Patient	Marcus [28]	2000–2011	CMR	0.91(0.88, 0.93)	0.80(0.76, 0.83)	37.69(26.00, 54.63)	–	indirect
			SPECT	0.83(0.73, 0.89)	0.77(0.64, 0.86)	15.84(9.74, 25.77)	–	
	Jaarsma [5]	1990–2010	CMR	0.89(0.88, 0.91)	0.76(0.73, 0.78)	26.42(17.69, 39.47)	0.91 (0.89, 0.93)	indirect
			SPECT	0.88(0.88, 0.89)	0.61(0.59, 0.62)	15.31(12.66, 18.52)	0.87 (0.85, 0.89)	
	Our	to 2013	CMR	0.79(0.72, 0.84)	0.75(0.65, 0.83)	11.10(5.73, 21.49)	0.84 (0.80, 0.87)	direct
			SPECT	0.70(0.58, 0.80)	0.76(0.66, 0.83)	7.19(4.68, 11.04)	0.79(0.76, 0.83)	

**Notes:** CT, coronary territory; CMR, cardiovascular magnetic resonance; SPECT, single photon emission computed tomography; –, no metion.

In this limited cohort of studies, the main results can be summarized as follows: 1). When assessed base on coronary territory, the diagnostic performance of perfusion-CMR assessed as SEN, SPE, and the area under the ROC curve (AUC) were superior over SPECT in detecting CAD. This findings of our study were in accord with the meta-analysis of Iwata *et al*. [27], which only included studies assessed base on coronary territory. 2). When assessed base on patient, the diagnostic performance of perfusion-CMR assessed as SEN and AUC values were superior over SPECT in detecting CAD, but inferior in SPE. Two previous indirect comparative meta-analyses demonstrated significantly higher SEN and DOR than SPECT but failed to show significant superiority in SPE (Table 2). As indirect comparative meta-analysis, we believe that might be caused by the different patients included in various studies. One may also speculate that may be caused by threshold effect with different diagnostic cut-off values, various data obtain and analysis methods. Such as, CE-MARC used a multiparametric CMR protocol, unlike other studies in our research, which used only the perfusion CMR components.

Compared with the result based on coronary territory, the diagnostic performace of CMR based on patient is relative low. We speculate that is related to the fact that perfusion CMR was more compared with the macroscopic coronary artery anatomy, but not assess, for example, collateral flow on the microvascular level [8].

As the numbers of comparative studies available in our research are relatively small, we did not assess factors such as different techniques or higher field strengths by subgroup analyses. New techniques and higher field strengths may make a further improvement in diagnostic accuracy. Jogiya *et al*. [30] reported that CMR showed a sensitivity, specificity, and diagnostic accuracy of 91%, 90%, and 91%, respectively, on patient basis and 79%, 92%, and 88% on coronary territory basis, with three-dimensional perfusion technique at 3 Tesla. It may offer CMR a more diagnostic performance over SPECT in future.

Some inherent limitations exist in our study and should be considered when interpreting our results. First, the number of comparative studies available in the literature and the sample size of these studies are relatively small, which is a particular problem in diagnostic studies [31]. These shortcomings may result in an overestimation of diagnostic accuracy, particularly in studies including non-representative samples of patients and invalid reference standards [29]. However, a systematic review [32], focused on meta-analysis studies from the Cochrane Database, showed that the number of studies eligible for meta-analysis is typically small in all medical areas and for all outcomes and interventions covered by the Cochrane Reviews. Second, six of ten studies did not enroll a consecutive or random sample of patients, which tended to be a risk of bias in patient selection. Third, publication bias may be of any meta-analysis. Our meta-analysis was based only on published studies, which are prone to report positive or significant results; the studies whose results are not significant or negative are often rejected or are not even submitted. Although it is suggested that the quality of the data reported in articles accepted for publication in peer-reviewed journals is superior to the quality of unpublished data [33], only including published studies may lead to reporting bias.

In conclusion, a limited number of studies demonstrated that CMR is more sensitive to detect CAD than SPECT in both on a per-patient basis and per-coronary territory, but inferior in specificity on a per-patient. In future, with new techniques and higher field strengths, large-scale, well-designed trials, are necessary to compare the diagnostic value of these two imaging techniques.

## Supporting Information

Checklist S1 **PRISMA 2009 checklist.**
(DOC)
